# YBX1 regulates tumor growth via CDC25a pathway in human lung adenocarcinoma

**DOI:** 10.18632/oncotarget.10080

**Published:** 2016-06-15

**Authors:** Shilei Zhao, Yan Wang, Tao Guo, Wendan Yu, Jinxiu Li, Zhipeng Tang, Zhenlong Yu, Lei Zhao, Yixiang Zhang, Ziyi Wang, Peng Wang, Yechi Li, Fengzhou Li, Zhe Sun, Yang Xuan, Ranran Tang, Wu-guo Deng, Wei Guo, Chundong Gu

**Affiliations:** ^1^ The First Affiliated Hospital & Institute of Cancer Stem Cell, Dalian Medical University, Dalian, China; ^2^ Department of Respiratory Medicine, The People's Hospital of Liaoning Province, Shenyang, China; ^3^ Lung Cancer Diagnosis and Treatment Center of Dalian, Dalian, China; ^4^ Sun Yat-Sen University Cancer Center, State Key Laboratory of Oncology in South China, Collaborative Innovation Center of Cancer Medicine, Guangzhou, China; ^5^ State Key Laboratory of Targeted Drug for Tumors of Guangdong Province, Guangzhou Double Bioproduct Inc., Guangzhou, China

**Keywords:** YBX1, CDC25a, cell cycle regulation, prognosis, lung adenocarcinoma

## Abstract

Y-box binding protein 1 (YBX1) is involved in the multi-tumor occurrence and development. However, the regulation of YBX1 in lung tumorigenesis and the underlying mechanisms, especially its relationship with CDC25a, was remains unclear. In this study, we analyzed the expression and clinical significance of YBX1 and CDC25a in lung adenocarcinoma and identified their roles in the regulation of lung cancer growth. The retrospective analysis of 116 patients with lung adenocarcinoma indicated that YBX1 was positively correlated with CDC25a expression. The Cox-regression analysis showed only high-ranking TNM stage and low CDC25a expression were an independent risk factor of prognosis in enrolled patients. High expression of YBX1 or CDC25a protein was also observed in lung adenocarcinoma cells compared with HLF cells. ChIP assay demonstrated the binding of endogenous YBX1 to the CDC25a promoter region. Overexpression of exogenous YBX1 up-regulated the expression of the CDC25a promoter-driven luciferase. By contrast, inhibition of YBX1 by siRNA markedly decreased the capability of YBX1 binding to CDC25a promoter in A549 and H322 cells. Inhibition of YBX1 expression also blocked cell cycle progression, suppressed cell proliferation and induced apoptosis via the CDC25a pathway in vitro. Moreover, inhibition of YBX1 by siRNA suppressed tumorigenesis in a xenograft mouse model and down-regulated the expression of YBX1, CDC25a, Ki67 and cleaved caspase 3 in the tumor tissues of mice. Collectively, these results demonstrate inhibition of YBX1 suppressed lung cancer growth partly via the CDC25a pathway and high expression of YBX1/CDC25a predicts poor prognosis in human lung adenocarcinoma.

## INTRODUCTION

During the past three decades, lung cancer has become the leading cause of cancer related deaths in world [1, 2]. Meanwhile, the incident of adenocarcinoma as the most aggressive histological type in lung cancer has been increasing rapidly [3]. In according to histological morphology and prognosis, the International Association for the Study of Lung Cancer (IASLC), the American Thoracic Society (ATS) and the European Respiratory Society (ERS) refined the lung adenocarcinoma multidisciplinary classification to provide essential references of individualized treatment in patients with lung adenocarcinoma [4]. Unfortunately, the five-year survival rate of lung adenocarcinoma still has no significant increased owing to early tumor metastasis and relapse [2, 5]. The poor prognosis has close relation with the features of deregulated proliferation and apoptosis resistance in adenocarcinoma [6, 7]. Therefore, investigating the mechanisms of malignant proliferation in lung adenocarcinoma has become considerably urgent.

The cell cycle rhythm disorder is one of the main culprits on malignant proliferation in adenocarcinoma [8, 9]. The cell cycle program is accurately controlled by activity of phosphorylate or dephosphorylate cyclin-dependent kinases (CDKs), such as CDK2, CDK4, and CDK6. CDC25a, a member of the Cdc25 dual phosphatase family, is a dual-specificity protein phosphatase which can dephosphorylate CDKs as the cell cycle checkpoint kinases [10]. Subsequently, dephosphorylated CDKs constitute a composition with cyclins protein, which phosphorylating Rb protein to demolish the repression of E2Fs activation leaded to cell cycle progression. Moreover, the composition is also a regulator of apoptosis attributed to inhibit p21 and p27 [11–13]. At present, high CDC25a expression has been reported in a variety of cancer cell lines or tumor tissues and has also related with tumorigenesis and poor prognosis [14–16]. From the previous literatures several transcriptional factors, such as Stat3 [17], Foxm1 [18], E2F [19], and CBP [20], have been detected to directly or indirectly switch on the activity of CDC25a promoter. Besides, some transcriptional suppressors, such as p21 [15] and Smad3/4 [21, 22], have been found to down-regulate CDC25a promoter activity. We speculate that if there are other transcription factors binding on its promoter that promote G1/S or G2/M entry and inhibit apoptosis. Therefore, it's essential to clarify how CDC25a is over-activated during malignant proliferation in lung adenocarcinoma.

The Y-box-binding protein 1 (YBX1), a 36 kDa multifunctional protein, can bind to the targets promoter with the so-called Y-box sequence (an inverted CCAAT box). YBX1 is a member of the cold-shock domain protein superfamily composed of three domains: the alanine/proline rich N-terminal domain, an S1 like cold shock domain and the large C-terminal domain [23, 24]. The last domain is the most important part which shuttled into nucleus from cytoplasm and bound to the promoter of targeting genes on the stimulation of hypoxia [25] or ultraviolet [26]. More importantly, a series downstream of YBX1 targeting genes are oncogenes which involved in malignant growth, chemotherapy resistance and tumor angiogenesis [27, 28]. Although YBX1 is exhibited as a poor prognostic factor in breast cancer, colon cancer, and ovarian cancer [29], it has no reported in lung adenocarcinoma by reference to new subtypes classification at present. There is a great number of researches have shown that YBX1 facilitates cell cycle progression and suppresses apoptosis in multiple cancer cell lines [30, 31]. Further, we previously detected the promoter of CDC25a (GenBank accession no. AJ242714.1) contained three Y-box sequences (Figure 4A) that might bind by YBX1. So we hypothesized that if YBX1 could bind to CDC25a promoter and up-regulate CDC25a expression to promote tumor cell overcoming cell cycle checkpoint restriction to satisfy unlimited malignant amplification.

Here we showed that the expression of CDC25a had close relationship with the expression of YBX1 in cancer tissues samples on the basis of novel adenocarcinoma subtypes classification by immunohistochemical staining for the first time. Furthermore, we investigated whether the expression of CDC25a was controlled by YBX1 in accordance with our hypothesis using ChIP and luciferase reporter gene assay. Simultaneously, knockdown of YBX1 by siRNA verified the down-regulation of cell cycle progression or apoptosis resistance in vitro and in vivo. Our study reveals that YBX1 activates CDC25a expression and suggests that the YBX1/CDC25a axis is involved in the cancer development and progression of human lung cancers.

## RESULTS

### Expression of YBX1 and CDC25a in lung adenocarcinoma tissues and cell lines

**Figure 1:**
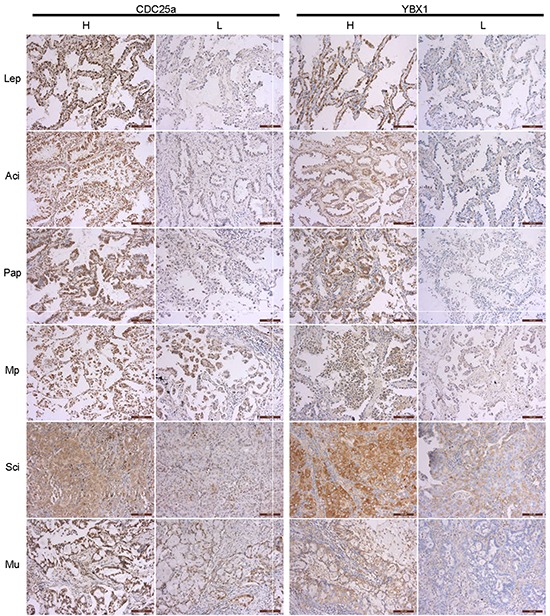
Immunohistochemical analyses of CDC25a and YBX1 proteins in different subtypes of lung adenocarcinoma The expression of CDC25a and YBX1 in MIA was detected by IHC assay. MIA, minimally invasive adenocarcinoma; Lep, lepidic predominant adenocarcinoma; Aci, acinar predominant adenocarcinoma; Pap, papillary predominant adenocarcinoma; Mp, micropapillary predominant adenocarcinoma; Sci, solid predominant adenocarcinoma; Mu, mucinous predominant adenocarcinoma.100X magnification of the micrographs as insert in the figure.

**Figure 2:**
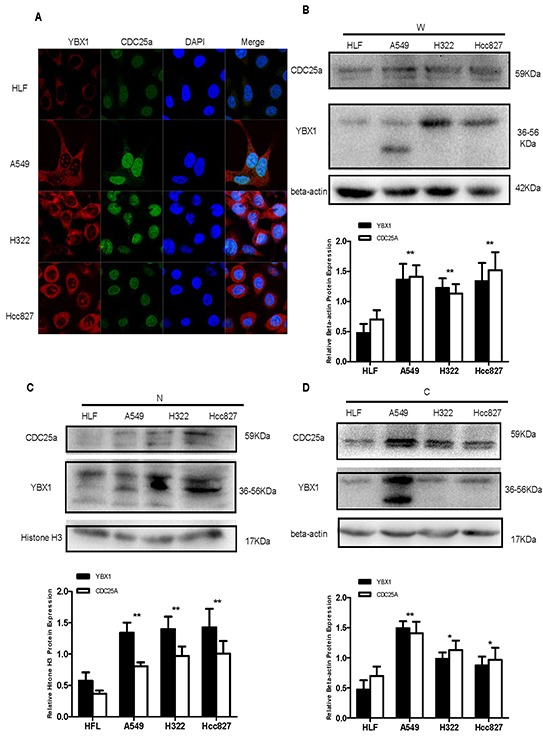
CDC25a and YBX1 were highly expressed in lung adenocarcinoma cell lines **A.** Co-localization of CDC25a and YBX1 in HLF, A549, H322 and Hcc827 cells by IF assay. Red: YBX1; Green: CDC25a. **B,C,D.** The expression of CDC25a and YBX1 proteins in total (W), nuclear (N) and cytoplasm (C) protein in lung adenocarcinoma cell lines and HLF cells detected by Western Blot. Expression of CDC25a and YBX1 were calculated relative to the Beta-actin or Histone H3 expression. The quantification of YBX1 and CDC25a was originated from all bands shown in the photograph. The date was repeated three times and presented as the mean±SD (*p<0.05, **p=<0.001 and # p>0.005).

We analyzed the expression of YBX1 and CDC25a in 116 patients with complete surgical resection of lung adenocarcinoma by IHC staining. Among the total patients, 56 (48.3%) patients had CDC25a high expression which predominantly located in nucleus and a small portion in cytoplasm. Meanwhile, 43 (37.1%) patients had YBX1 high expression which was usually seen in cytoplasm and a little in nucleus or extracellular matrix (Figure 1). More interestingly, the patients with MIA, a less aggressive subtype of adenocarcinoma, had neither YBX1 nor CDC25a high expression in accordance with IASLC/ATS/ERS international multidisciplinary classification. Similarly, the higher pathological component risk rating, the more proportion of YBX1 and CDC25a expression in tumor lesions. Subsequently, we explored the expression and location of YBX1 and CDC25a in vitro by immunofluorescence assay (Figure 2A). YBX1 was mainly presented in cytoplasm of human lung adenocarcinoma cell lines (A549, H322 and Hcc827) but little in normal human embryonic lung fibroblasts cell line (HLF). Moreover, A549, H322 and Hcc827 cells had obvious YBX1 nuclear translocation compared with HLF cells. CDC25a was also found to be highly expressed in nucleus of A549, H322 and Hcc827 cells. The same result was confirmed using the total, nuclei and cytoplasm proteins extracted from HLF, A549, H322 and Hcc827 cells by Western blot analysis (Figure 2B–2D).

### Relationship between YBX1 or CDC25a expression with clinicopathologic factors in lung adenocarcinoma and survival of patients

**Figure 3:**
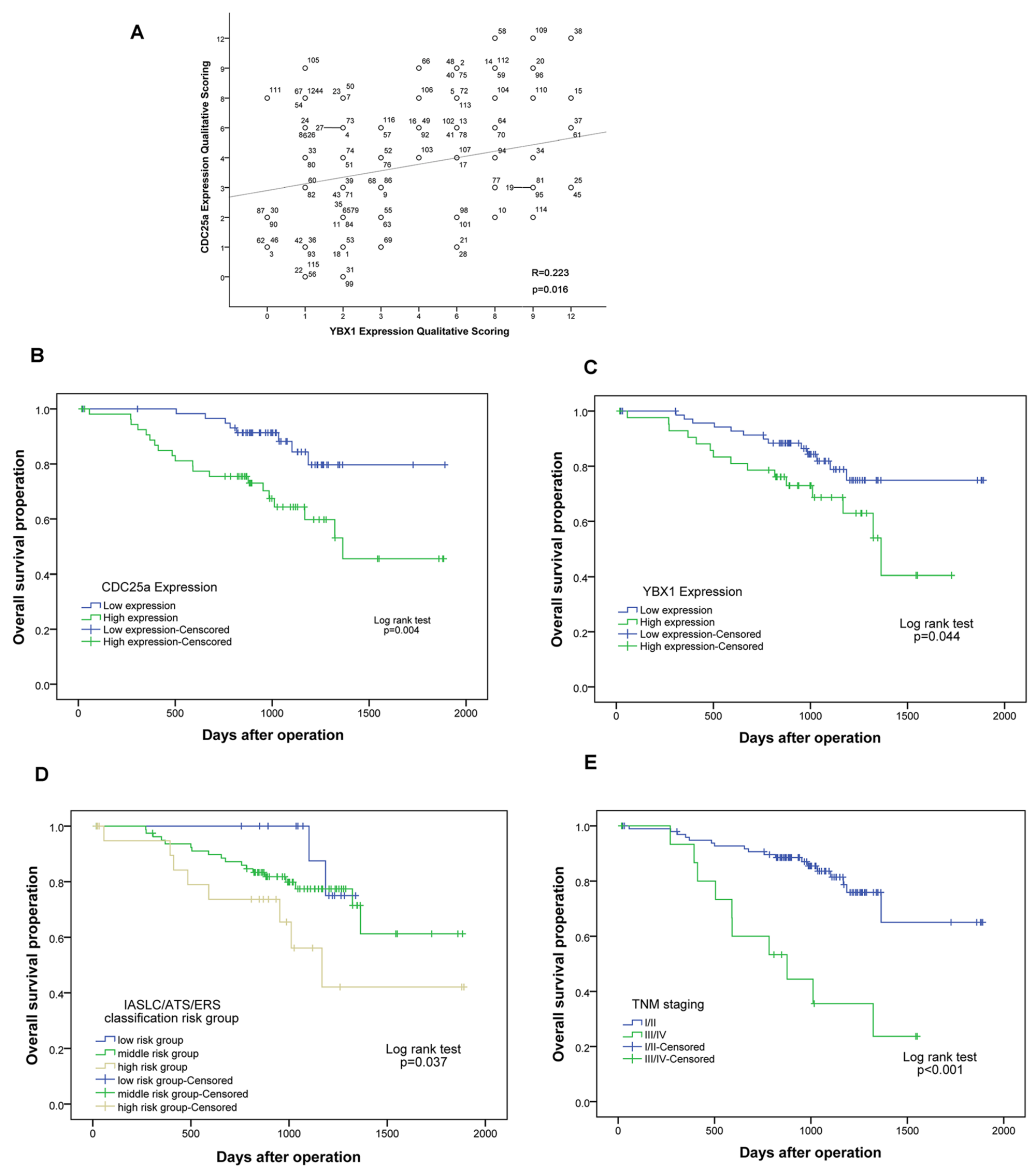
The scattered/correlation blot between CDC25a and YBX1 expression and survival curves of CDC25a/YBX1 expression, IASLC/ATS/ERS classification and TNM stages in 116 patients with lung adenocarcinoma **A.** A scattered/correlation blot between CDC25a and YBX1 expression in 116 patients. Each number represents a patient. **B.** The overall 5-year survival rates in the patients with low CDC25a expression and high CDC25a expression were 79.7% and 45.5%, respectively (p=0.004). **C.** Survival curves of low and high expression of YBX1 was 74.9% and 40.5%, respectively (p=0.044). **D.** The 5-year survival rates of low, middle and high risk classification risk group was 75.0%, 61.3% and 42.1%, respectively (p=0.037). **E.** The 5-year survival rates of low TNM stage (I/II) and high TNM stage (III/IV) was 65.0 and 23.7%, respectively (p<0.001).

The clinicopathologic characteristics of the patients were summarized in Table 1. The expression of CDC25a was significantly associated with IASLC/ATS/ERS classification of risk groups, differentiation, lymph node metastasis and TNM stage (p<0.001, p=0.007, p=0.026, p=0.033, respectively), but no not sex, ages, pleural invasion, distant metastasis, T state, operation mode and CEA. However, YBX1 expression only has relationship with T state (p=0.006) and TNM stage (p=0.006). YBX1 had positive correlation with CDC25a (p=0.016, R=0.223) shown in Table 2 (Figure 3A).

**Table 1:** Relations between the level of CDC25a or YBX1 expression and clinicopathologic characteristics in lung adenocarcinoma

Characteristics	CDC25a		P-value	YBX1		P-value
	High(%)	Low		High(%)	Low	
Over all	56(48.3)	60		43(37.1)	73	
Sex			0.887			0.971
male	25(49.0)	26		19(37.3)	32	
female	31(47.7)	34		24(36.9)	41	
Age			0.816			0.726
≤64y	32(49.2)	33		25(38.5)	40	
>64y	24(47.1)	27		18(35.3)	33	
IASLC/ATS/ERS classification risk group (predominant adenocarcinoma)			<0.001			0.156
low risk	1(7.1)	13		2(14.3)	12	
MIA	0(0.0)	3		0(0.0)	3	
lepidic	1(9.1)	10		2(18.2)	9	
middle risk	38(47.5)	42		33(41.2)	47	
papillary	14(42.4)	19		15(45.5)	18	
acinar	24(51.1)	23		18(38.3)	29	
high risk	17(77.3)	5		8(36.4)	14	
micropapillary	5(1.3)	1		2(33.3)	4	
soild	3(75.0)	1		2(50.0)	2	
mucinous	7(70.0)	3		3(30.0)	7	
others(fetal and signet-ring type)	2(100.0)	0		1(50.0)	1	
Differentiation			0.007			0.292
well	25(36.2)	44		22(31.9)	47	
moderate	16(64.0)	9		10(40.0)	15	
poor	15(68.2)	7		11(50.0)	11	
Pleural invasion			0.115			0.139
absent	37(54.4)	31		29(42.6)	39	
present	19(39.6)	29		14(29.2)	34	
Distant metastases			0.140			0.063
with	54(47.4)	60		41(36)	73	
without	2(100.0)	0		2(100.0)	0	
Lymph node metastasis			0.026			0.732
with	17(68.0)	8		10(40.0)	15	
without	39(42.9)	52		33(36.3)	58	
T states			0.060			0.006
T1	15(36.6)	26		9(22.0)	32	
T2	36(52.2)	33		29(42.0)	40	
T3/T4	5(83.3)	1		5(83.3)	1	
TNM Stage			0.033			0.006
I	16(36.4)	28		9(20.5)	35	
II	27(49.1)	28		24(43.6)	31	
IIIA	10(71.4)	4		7(50.0)	7	
IIIB/IV	3(100.0)	0		3(100.0)	0	
Operation mode			0.076			0.340
wedge resection	4(26.7)	11		8(53.3)	7	
lobectomy	50(50.5)	49		34(34.3)	65	
pneumonectomy	2(100.0)	0		1(50.0)	1	
Concentration of CEA (ug/l)			0.391			0.739
<5	40(46.0)	47		33(37.9)	54	
≥5	16(55.2)	13		10(34.5)	19	

**Table 2:** The positive correlation between CDC25a and YBX1 protein expression in lung adenocarcinoma specimens

	CDC25a		P-value	R-value
	High	Low		
YBX1				
High	27	29	0.016	0.223
Low	16	44		

Furthermore, we analyzed the relationship between clinicopathologic factors and prognosis in 116 patients with lung adenocarcinoma. Kaplan Meier analysis showed patients with high IASLC/ATS/ERS classification of risk groups (p=0.037), poor differentiation (p=0.001), lymph node metastasis (p=0.008), abnormal CEA (p=0.045), high CDC25a expression (p=0.004), high YBX1 expression (p=0.044) or uplifted TNM stage (p<0.001) had poorer survival outcome by log-rank test (Table 3 and Figure 3B-3E). The multivariate Cox proportional hazards model analysis of risk factors showed that high TNM stage (HR=3.428, 95%CI: 1.519-7.737, p=0.003) and low CDC25a expression (HR=2.384, 95%CI: 1.008-7.642, p=0.048) were independent prognostic risk factors in lung adenocarcinoma but no high YBX1 expression (HR=1.211, 95%CI: 0.496-2.952, p=0.674) (Table 4). Five-year over-survival of low CDC25a expression was 79.7% significantly longer than high expression (45.5%) using the Kaplan Meier method (Figure 3B).

**Table 3:** The five-year overall survival on different clinicopathological factors was used Kaplan Meier and univariate analysis

Variable	5-OS (%)	log-rank test P-value	Univariate analysis	
			HR	95%CI
Sex		0.345		
male	74.1		1	
female	53.3		1.445	0.670-3.114
Age		0.611		
≤64y	64.0		1	
>64y	49.5		1.218	0.569-2.606
IASLC/ATS/ERS classification risk group (predominant adenocarcinoma)		0.037		
high risk	42.1		1	
middle risk	61.3		0.470	0.204-1.082
low risk	75.0		0.250	0.053-1.188
Differentiation		0.001		
poor	26.0		1	
moderate	49.0		0.951	0.393-2.301
well	79.4		0.238	0.092-0.619
Pleural invasion		0.744		
present	64.7		1	
absent	60.3		0.882	0.414-1.879
Distant metastases		0.066		
without	69.1		1	
with	50.0		5.404	0.717-40.723
Lymph node metastasis		0.008		
without	49.7		1	
with	49.1		2.711	1.263-5.822
T states		0.101		
T3/T4	33.3		1	
T2	58.9		0.262	0.087-0.791
T1	68.0		0.226	0.067-0.762
TNM Stage		<0.001		
I/II	65.0		1	
III/IV	23.7		4.538	2.076-9.920
Operation mode		0.933		
wedge resection	73.3		1	
lobectomy/other	54.7		1.047	0.362-3.027
Concentration of CEA (ug/l)		0.045		
<5	60.6		1	
≥5	61.7		2.185	0.998-4.780
YBX1		0.044		
low	74.9		1	
high	40.5		2.111	1.003-4.444
CDC25a		0.004		
low	79.7		1	
high	45.5		3.124	1.370-7.123

**Note:**

**Abbreviations:** 5-OS, five-year overall survival; CEA, carcino-embryonic antigen.

**Table 4:** Multivariate analysis of overall survival used Cox-regression

Variable	Multivariate analysis		P-value
	HR	95%CI	
Differentiation			
poor	1		
moderate	0.962	0.396-2.339	0.932
well	0.404	0.139-1.176	0.097
Lymph node metastasis			
without	1		
With	1.117	0.365-3.417	0.846
T states			
T3/T4	1		
T2	0.774	0.182-3.289	0.728
T1	0.508	0.142-1.822	0.299
TNM Stage			
I/II	1		
III/IV	3.428	1.519-7.737	0.003
YBX1			
Low	1		
High	1.211	0.496-2.952	0.674
CDC25a			
low	1		
high	2.384	1.008-7.642	0.048

### Regulation of CDC25a promoter activation by YBX1 in lung adenocarcinoma

**Figure 4:**
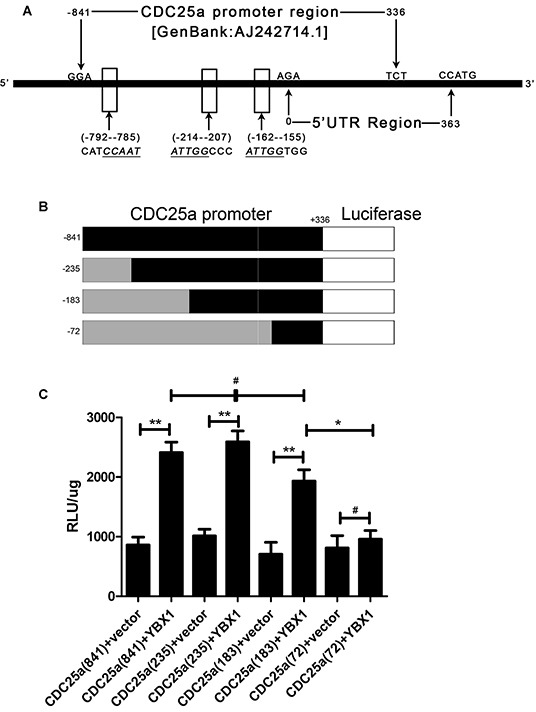
YBX1 bound to CDC25a promoter region and positively regulated its transcriptional activation in lung adenocarcinoma cells **A.** Three YBX1-binding possibility regions (indicated by the arrows) were checked in CDC25a promoter (GENE BANK: AJ242714.1). **B.** A 5′-flanking DNA fragment from position −841 to +336 (−841/+336, −235/+336, −183/+336, −72/+336) of human CDC25a gene was constructed into a promoter less luciferase expression vector pGL3. **C.** A549 cells were cotransfected with YBX1 and CDC25a promoter-driven luciferase plasmids for 48 hrs. The proteins were extracted, and luciferase activity was detected by luciferase reporter assay kit. pCDNA3.1-vector were negative control. Luciferase activity was detected.

**Figure 5:**
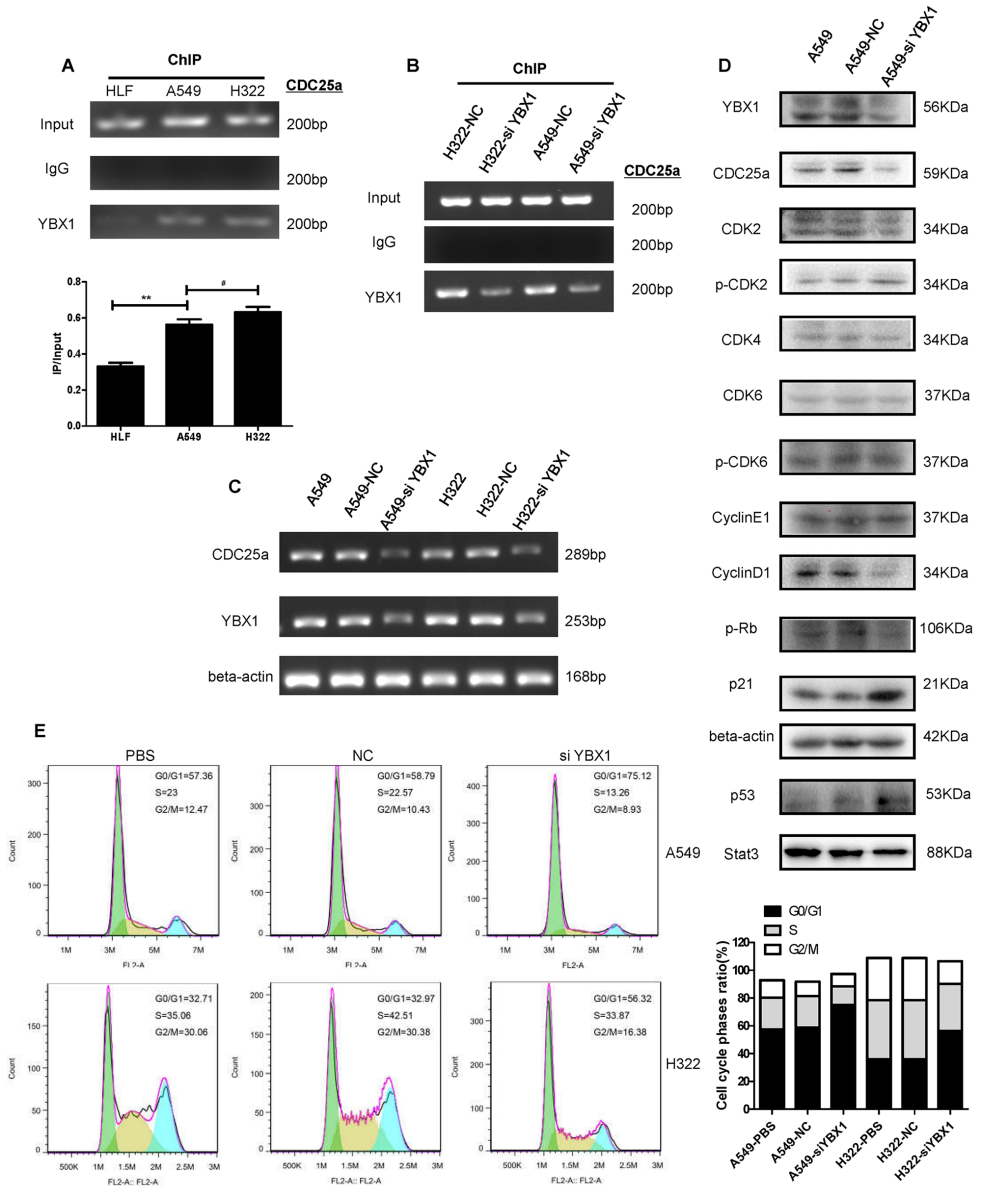
YBX1 knockdown inhibited the expression of CDC25a and changed its downstream in G1/S checkpoint pathway **A.** In accordance with luciferase activity, the ChIP assays were performed in HLF, A549 and H322 cells to confirm the endogenous of YBX1 binding to the CDC25a promoter (−352 to −153 bp) using an anti-YB-1 antibodyor normal rabbit IgG. **B.** YBX1 knockdown reduced endogenous YBX1 binding to the CDC25a promoter using ChIP assays in A549 and H322 cells. **C.** RT-PCR assay revealed si-YBX1 inhibited the mRNA expression of YBX1 and CDC25a in A549 and H322 cells. **D.** G1/S checkpoint pathway was detected by Western blot assay in A549 cells transfected with si-YBX1. **E.** Thesi-YBX1-transfected A549 and H322 cells stagnated in G0/G1 phase by cell cycle analysis.

To clarify the possible role of YBX1 in the regulation of CDC25a, we searched and found that CDC25a promoter region (GeneBank: AJ242714.1) had several inverted CCAAT box which maybe bound and activated by YBX1 during its transcription (Figure 4A). To verify the regulation of CDC25a by YBX1, the lung cancer A549 cells were co-transfected with YBX1 overexpressing vector and a luciferase reporter driven by the four different lengths of CDC25a promoter (Figure 4B). The results showed that the luciferase expression was higher in cells co-transfected with YBX1 overexpressing vector/CDC25a (−841/+336, −235/+336 and −183/+336)-luciferase plasmids than those co-transfected with YBX1 overexpressing vector/CDC25a (−72/+336)-luciferase plasmids or the negative control (pCDNA3.1-vector)/corresponding CDC25a-luciferase plasmids (Figure 4C). Moreover, ChIP assay was also performed in HLF, A549 and H322 cells (Figure 5A) and its illustrated that YBX1 could activate the transcription of CDC25a and revealed YBX1 could bound to the designated regions of CDC25a promoter(−235/−183).

### Regulation of G1/S transform by YBX1 via CDC25a pathway

ChIP assay shown that the less endogenous YBX1 bond to CDC25a promoter region in siYBX1-transfected A549 and H322 cells than those transfected with the control siRNA and mRNA of YBX1 or CDC25a in the si-YBX1-treated cells was declined (Figure 5B, 5C). Next, we extracted the total protein from the PBS-A549, NC-A549 and siYBX1-A549 cells, and investigated whether knockdown of YBX1 could suppress the expression profiles of genes related to G1/S transform in cell cycle of CDC25a pathway. Expression of CDC25a, p-RB, Stat3 and cyclin D1 was apparently down-regulated in siYBX1-A549 cells, but the expression of other related genes like CDK2, CDK4, CDK6 and cyclinE1 had no obvious changes by YBX1 knockdown. However, the expression of p21 and p53, the cell cycle inhibitor proteins, was up-regulated in siYBX1-A549 cells (Figure 5D). To further verify the effect of siYBX1 on cell cycle, we used flow cytometric analysis and observed the decreased population of cells in S phase by YB-1 knockdown in A549 and H322 cells (Figure 5E). The result indicated that si-YBX1 partly inhibited the downstream of CDC25a in cell cycle.

### Regulation of proliferation, apoptosis and migration by YBX1 in lung adenocarcinoma in vitro

**Figure 6:**
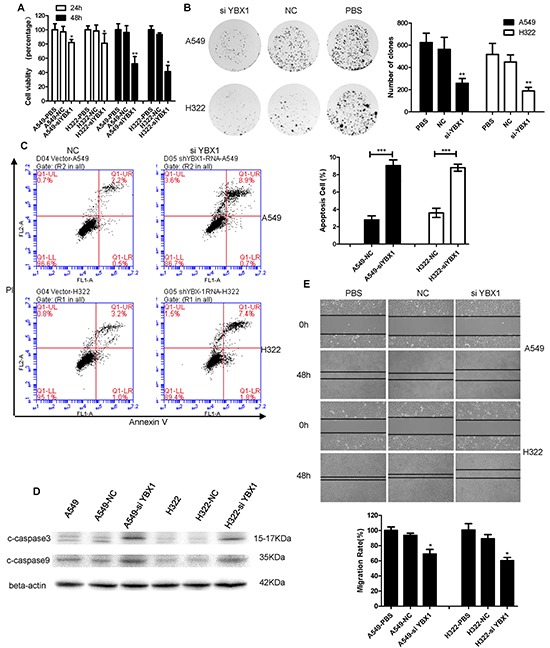
YBX1 knockdown inhibited the growth and migration of lung adenocarcinoma cells and induced apoptosis **A.** Cell viability was analyzed by MTT in A549 and H322 cells transfected with si-YBX1 at 24 and 48h. The effect on cell viability was assessed as the percent cell viability compared with control-PBS group, which were arbitrarily assigned 100% viability. **B.** Colony formation assay of A549 and H322 cells transfected with si-YBX1 and its quantification. **C.** A549 and H322 cells were treated with si-YBX1 and the apoptosis was determined by a FACS analysis. **D.** Cleaved caspase-3/9 proteins in A549 and H322 cells were respectively analyzed by Western blot. **E.** Cell migration was analyzed by a wound-healing assay. A549 and H322 cells were seeded in 6-well plates and grown to full confluence. Migration rate was calculated as MR=[initial gap(0h)- terminal gap(48h)/initial gap(0h)]100%. The date was repeated three times and presented as the mean±SD (*p<0.05,**p=<0.001 and# p>0.005).

The cell cycle of malignant tumor was closely related with tumor intensive proliferation and subdued apoptosis. We next examined the effect of YBX1 on the cell viability by MTT and clone formation assay. As shown in Figure 6A, the knockdown of YBX1 in both A549 and H322 cells significantly suppressed cell viability compared with the cells treated with the control siRNA or PBS. Consistent with the results from the inhibition of clone formation, the siYBX1-treated cells formed nearly 200 clones after 7 days' growth less than other agent-treated cells (Figure 6B). We also determined the effect of YBX1 on apoptosis by flowcytometric analysis (Figure 6C). The results showed that after transfected siYBX1 48 hours, the number of A549 and H322 apoptotic cells was less than the control groups. To further make sure the effect of YBX1 on cell apoptosis, we detected the expression of the apoptosis proteins, cleaved-caspase-3 and cleaved-caspase-9, in A549 and H322 cells by Western blot analysis (Figure 6D). Knockdown of YBX1 cells up-regulated the expression of the cleaved-caspase-3 and cleaved-caspase-9 as compared with the control groups. In addition, the wound-healing assay also revealed the inhibition effect of the siYBX1 on tumor cell mobility in A549 and H322 cells. As shown in Figure 6E, the wounding space between cell layers was significantly reduced in the control siRNA- or PBS-treated A549 cells after 48 hours compared with the siYBX1-treated cells. These results suggest that YBX1 has the properties in regulating cell proliferation, apoptosis and migration in lung adenocarcinoma cells.

### Inhibition of tumor growth by YBX1 siRNA in a lung cancer xenograft mouse model in vivo

**Figure 7:**
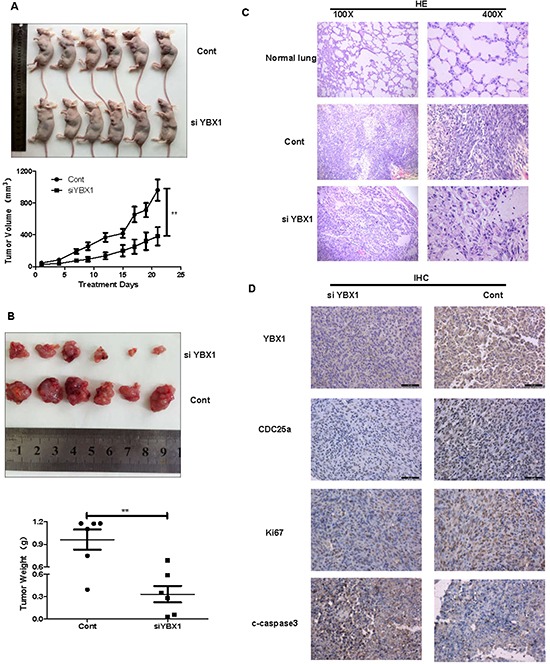
YBX1 knockdown inhibited tumor growth by down-regulating CDC25a expression **A.** A photo of tumor grafts in nude mice injected with non-specific control siRNA (n=6) or si-YBX1 (n=6) 21 days latter and tumor volume of each group of nude mice was measured by V=(width^2^×length)/2; **P<0.01. **B.** The morphology of tumor xenografts in each nude mice after anatomy at 21 days of treatment was shown and tumor weight was analyzed by electronic scales; **P<0.01. **C.** H&E staining and **D.** Immunohistochemical analysis of YBX1, CDC25a, Ki 67 and cleaved caspase 3 protein expression in tumor samples.

To determine whether YBX1 could be a novel molecular therapeutic target for tumor growth in nude mice with human lung adenocarcinoma xenografts, the lung adenocarcinoma A549 cells were injected in mice hypodermis, and the siYBX1 and the control siRNA were treated in the corresponding groups. As shown in Figure 7A and 7B, the smaller tumor volume and lighter weights were detected in the siYBX1-treated mice. H&E staining also showed that the siYBX1-treated tumor cells showed little cell pleomorphism, including pleomorphic giant nucleu, obvious nucleoli and karyokinesis, compared with the control siRNA-treated mice (Figure 7C). Furthermore, we also showed that inhibition of YBX1 suppressed the expression of CDC25a in tumor tissues by IHC analysis as well as Ki 67 and stimulated cleaved caspase 3 expression (Figure 7D). These results demonstrated that YBX1 activate the growth of the xenografted human lung adenocarcinoma partly through CDC25a pathway in vivo.

## DISCUSSION

Previous studies have shown that both YBX1 and CDC25a participated in the regulation of cell cycle and apoptosis, so it was important to investigate whether there was hidden affiliation between YBX1 and CDC25a. In our present study, IHC staining was performed to show the expression of CDC25a and YBX1 in 116 patients with lung adenocarcinoma. But strangely, the subcellular localization of YBX1 was primarily appeared in cytoplasm unlike previous literature. This might be because nuclear antigen was blocked in process of tissue fixation by formalin, which could seal off relatively faint antigen. However, all human lung adenocarcinoma cell lines (A549, H322 and Hcc827) expressed more obvious nucleus shifting of YBX1 compared with HLF cells using immunofluorescence. In addition, the expression of YBX1 and CDC25a from lung adenocarcinoma cells' total, nuclear and cytoplasm protein was also higher than HLF cells analyzed by Western blot assays. However, YBX1 in different cells had multi-bands maybe due to post-translation cleavage or relative charge under different metabolic state. We further discovered positive correlation between YBX1 and CDC25a expression (R=0.223, p=0.016) in human lung adenocarcinoma. The most exciting thin is three inverted CCAAT boxes, probable being bound by YBX1, were chanced upon in CDC25a promoter region. Based on all of the clues related to YBX1 and CDC25a, we hypothesized that YBX1 functioned as transcription factor and bound to CDC25a promoter to participate the transcription level of CDC25a, and also accelerate the process of cell cycle. Next, we certified the assumptive mechanisms that YBX1 as the transcriptional activator to accelerate expression of CDC25a, and then the cell cycle, growth and apoptosis in human lung adenocarcinoma cells as well as the inhibition of YBX1 on the growth of xenografts in mice were tested.

In our study, we assessed the expression of YBX1 and CDC25a in patients with complete with surgical resection of lung adenocarcinoma on basis of IASLC/ATS/ERS international multidisciplinary classification for the first time. High proportion of CDC25a expression in patients was increased following with the advance of pathological risk rating, but YBX1 expression (p= 0.156) had not displayed the association with pathological risk groups, suggesting that YBX1 expression maybe depend on the subtypes of adenocarcinoma or the number of high risk group was small (n=22). As above mentioned, significant consistency between YBX1 and CDC25a expression in patients' specimens as well as in vitro was found.

Then we demonstrated the hypothesis that CDC25a transcription activation was dependent on YBX1, which provided a new perspective mechanism on CDC25a expression regulation in lung adenocarcinoma. The overexpression of YBX1 significantly increased CDC25a promoter-driven luciferase gene expression in A549 cell line. Furthermore, we showed that endogenous YBX1 bound to the specific CDC25a promoter region in lung adenocarcinoma cells as compared to the HLF cells by ChIP assay. On the contrary, knockdown of YBX1 expression could reduce the binding of YBX1 on the CDC25a promoter in A549 and H322 cells using ChIP assays (Figure 5B). This further explained YBX1 could up-regulate CDC25a promoter transcriptional activity directly.

YBX1, a potent mitogenic biomarker, was reported to participate in the regulation of CDC6 and cyclin D transcription [27, 37], indirectly influencing the related protein activity in G1-S transition of cell cycle. However, there was no significant difference among the siYBX1-A549, the control siRNA-A549 and PBS-A549 cells, this maybe due to the tissue specificity. Under the CDC25a pathway, the down-regulation of CDC25a, cyclinD1, Stat3 and p-Rb and the up-regulation of p21, p53 and p-CDK2 were observed by YBX1 knockdown in A549 cells. The data further confirmed the original ideas that YBX1 directly or indirectly activated CDC25a to affect activity of phosphorylate CDKs such as p-CDK2, and also increased cyclinD1 [27]. Flowcytometric analysis farther illustrated G1/S arrest of cell cycle in A549 and H322 cell lines. Subsequently, we found that knockdown of YBX1 by siRNA significantly decreased CDC25a activity and inhibited growth in vitro and in vivo, which indicated the key role of YBX1/CDC25a axis involved in lung cancer development and progression. Simultaneously, high expression of YBX1 or CDC25a in 116 patients with lung adenocarcinoma had shorter 5-year overall survival than low expression (5-OS%:40.5% VS 74.9%, p=0.044 and 5-OS%:45.5% VS 79.7%, p=0.004, respectively). The multivariate analysis of risk factors showed that high TNM stage (HR=3.428, 95%CI: 1.519-7.737, p=0.003) and low CDC25a expression (HR=2.384, 95%CI: 1.008-7.642, p=0.048) were independent prognostic risk factors in lung adenocarcinoma but not YBX1. The unbalanced selection of patients (I stage: n=44; II stage: n=55) at early stage in enrolled crowd maybe cause the statistical no significance of YBX1 expression [36, 38], and the further study will be investigated in our subsequent research.

In summary, YBX1 increases the promoter activity and expression of CDC25a in human lung adenocarcinoma cells. Knockdown of YBX1 arrested the G1/S transition of cell cycle and further suppressed lung tumor growth in vitro and in vivo. High CDC25a expression and TNM stage were significantly independent factors for predicting poor prognosis of patients with lung adenocarcinoma. All the evidence showed that the novel YBX1/CDC25a pathway is a potential therapeutic target for the treatment of human lung adenocarcinoma.

## MATERIALS AND METHODS

### Cell lines and cell culture

Three human lung adenocarcinoma cell lines (A549, H322 and Hcc827) and one normal human embryonic lung fibroblasts cell line (HLF) were obtained from American Type Culture Collection (ATCC, Manassas, VA). All Cells were cultured in ATCC-recommended medium supplemented with 10% fetal bovine serum (FBS) and maintained at 37°C in a humidified atmosphere containing 5% CO_2_.

### Antibodies and other reagents

The primary antibody against YBX1 was purchased from Abcam (Cambridge, UK). Other primary antibodies, such as CDC25a, CDK2, p-CDK2, CDK4, CDK6, p-CDK6, cyclin D1, cyclin E1, p-RB, and p21 were got from Santa Cruz Biotechnology (Santa Cruz, CA, USA). Besides, beta-actin, Ki-67, p53, Stat3, cleaved caspase-3 and cleaved caspase-9 antibodies were obtained from Protein-Tech (Protein-Tech Group Inc., Chicago, IL, USA). Trypsin, steptavidin-agarose beads and other chemicals were purchased from Sigma Chemical Co. (St. Louis, MO) unless special version.

### Protein extraction and western blot analysis

Collect cells at optimum cell culture time in a microcentrifuge tube. Total, nuclei or cytoplasm protein was collected as what we did in previous article [32]. The concentration of the total, nuclei or cytoplasm cell proteinlysate was quantified by BCA protein assay kit (Thermo Fisher Scientific). Then 30-50g of protein lysates were separated by 8-12% SDS-PAGE and transferred to a polyvinylidene difluoride (PVDF) membrane. The membrane was incubated with the specific antibodies (appropriate concentration refered to specification) and protein band was detected with enhanced chemiluminescence system.

### Immunofluorescence and confocal microscopy

The grown cells were seeded on coverslip within 6-well plates. The coverslip was slightly taken out and washed with PBS, fixed with 4% paraformaldehydeb for 30 mins at room temperature, quenched with 0.2% TritonX-100 for 15 minutes, and then blocked with 10% bovine serum albumin in PBS for 30 minutes. The primary antibodies against YBX1 and CDC25a diluted in blocking solution incubated overnight at 4°C. Non-immune IgG was used as control. After being washed with PBS three times, secondary fluorescein isothiocyanate or tetra methyl rhodamine isothiocyanate-conjugated antibodies was added for 1 h. Washed slides with PBS once again and stained 4′, 6-diamidino-2-phenylindole (DAPI) (Beyotime, China) for 5 min to counterstain cell nuclei. The location of YBX1 and CDC25a protein in cells was exhibited with a Leica DM 14000B confocal microscope.

### Plasmid vector and small interfering RNA

ShangHai GenePharma Co (Shanghai, China) supplied three siRNAs targeting YBX1 and nonspecific siRNA (sense: 5′-UUCUCCGAACGUGUCACGUTT-3′; antisense: 5′-ACGUGACACGUUCGGAGAATT-3′), but only one siRNA for YBX1 (sense: 5′-GGUUCCCAC CUUACUACAU-3′; antisense: 5′-AGAAGGUCAUCG CAACGAA-3′) can knockdown YBX1 effectively, hereafter this siYBX1 was used in the whole study. The overexpression vector of YBX1 (pcDNA3.1-YBX1) and control vector (pcDNA3.1-vector) plasmids were designed and synthesized by Cyagen (Cyagen Biosciences Inc., China). The different nucleic acid alignments of CDC25a promoter (−841 to +336, Gene-Bank: AJ242714.1) were synthesized by Wanze (Wanze Biosciences Inc., China) and then recombinant plasmid vectors pGL3-CDC25a (−841/+336, −235/+336, −183/+336 and 72/+336)) expressing luciferase were constructed in our lab, and used in the transfection experiments.

### Transient transfection of lung adenocarcinoma cells

Human lung adenocarcinoma cell lines (A549, H322) were transfected with siYBX1 or nonspecific siRNA (1-2 μg) mediated by Lipofectamine 2000 (Invitrogen, Carlsbad, CA) as well as pcDNA3.1-YBX1 or pcDNA3.1-vector in 6-well plates (2X10^4^ cells per well). After treating 48 hours, protein or cells were gathered to test and verify by requisite assays.

### Analysis of CDC25a promoter activity

Cells were plated into 6-well plates and transfected with different recombinant plasmid vectors pGL3-CDC25a (1ug/well) and simultaneously co-transfected with either pcDNA3.1-YBX1 or pcDNA3.1-vector. After 48 hours treatment, the expressed luciferase activity was measured by using a DUAL-luciferase reporter assay kit (BioVision, Inc.CA, USA). Transfection efficiency was normalized by co-transfection with Renilla luciferase reporter.

### Chromatin immunoprecipitation assay (ChIP)

Genomic DNA was collected from A549, H322, HLF cells or siRNA-YBX1 treated A549, H322 cells and fragmented by ultrasound. The ChIP assay was performed using the ChIP IT Express kit (Active Motif, Rixensart, Belgium) following the manufacturer's instructions. After the elution, the DNA was subjected to PCR to amplify a 200 bp region (−352 to −153 bp) of the CDC25a promoter using the primers (sense: 5′-AGGTTCTGCTGGGAGTTT-3′ and antisense: 5′-GAATCCACCAATCAGTAAGC-3′). The PCR products were separated on a 1% agarose gel and visualized by ethidium bromide staining, and then the band density was measured through quantitative analysis.

### Patient characteristics and immunohistochemistry staining

Primary tumor specimens from 116 patients (median age: 64-year; range 45 from 86 years) with complete surgical resection of lung adenocarcinoma were consecutively obtained at the First Affiliated Hospital of Dalian Medical University during January 2008 and December 2010. The patients received chemotherapy or radiotherapy prior to the operation was excluded. The 7th edition International Union Against Cancer/American Joint Committee on Cancer TNM classification [33] was applied to all enrolled patients. Histologic classification of each slide was independently examined by two pulmonary pathologists (Dr. Sun and Dr. Gu) based on IASLC/ATS/ERS international multidisciplinary classification of lung adenocarcinoma [4], The new classification included: (1) adenocarcinoma in situ (AIS), (2) minimally invasive adenocarcinoma(MIA), (3) acinar(Aci), (4) papillary(Pap), (5) micropapillary(MP), (6) solid(Sol), (7) mucinous(Mu). The follow-up of patient was according to our previous process and its ranging from 3 to 60 months after the primary operation (median follow-up time 43.2 months). The study was approved by the Medical Ethical Committees of the First Affiliated Hospital of Dalian Medical University.

All resected specimens were obtained from primary lesions, fixed with formalin, embedded with paraffins, serial 4 μm sections were prepared. The sections were briefly incubated with xylene, rehydrated with graded ethanol solutions, incubated with methyl alcohol containing 3% hydrogen peroxide and immersed in a citrate buffer for antigen retrieval. Immunohistochemistry (IHC) staining was performed using Streptavidin-Peroxidase IHC assay kit (ZSGB-bio, China) following the manufacturer's instructions. Antibodies of polyclonal YBX1 and monoclonal CDC25a antibody diluted 1:200 and 1:100 in PBS containing 2% goat bovine serum respectively. Immunostaining was evaluated by two pulmonary pathologists using a blind protocol design. For each specimen, the total score of intensity expression (negative staining: 0 point; weak staining: 1 point; moderate staining: 2 point; and strong staining: 3 point) multiplying stained cell numbers (positive cells as≤25% of the cells: 1 point; 26-50% of the cells: 2 point; 51-75% of the cells: 3 point; >75% of the cells: 4 point) of YBX1 or CDC25a was estimated. When the sample was scored ≥6 point, we defined it as high expression, otherwise low expression [34]. The positive control of YBX1 and CDC25a was refered to ovarian cancer and prostate cancer respectively from proteinatlas website (http://www.proteinatlas.org).

### Reverse transcription-polymerase chain reaction (RT-PCR)

Total RNA was isolated from cultured cells using Trizol Reagent (TaKaRa Bio.) according to the manufacturers' instructions. Reverse transcription was carried out with TIANScript II RT Kit (TIAN GEN.) The PCR primers sequences sequences were listed as follows: for YBX1 (sense:5'-CAATGTAAGGAACGGATATGG-3′, antisense:5'-TTCC CCACTCTCACTATTCTG-3′),CDC25a (sense:5'-CGCG TCCCTGAACCGCGGAG-3′, antisense:5'-CGGCGGCT GAA GCGCCAAATA-3′),and GAPDH (sense:5'-GGC ACCCAGCACAATGAA-3′, antisense:5'-TA GAAGCA TTTGCGGTGG-3′). The samples were denatured at 94°C for 3 min and performed 30-40 cycles of PCR amplification as follows: denature 94°C for 30 s, anneal 55°C-60°C for 15-30s, and extend 68°C for 1min. Maintain the reaction at 4°C after cycling or be stored at −20°C until use.

### Clone formation assay

To analyze the effect of YBX1 on tumor clonogenicity, colony formation assay was performed in vitro. A549 and H322 cells were seeded in 6-well plates (500 cells per well) and exposed to siYBX1, control siRNA or PBS respectively. The colonies were maintained in a 37°C, 5% CO_2_ incubator for 14 days followed with purification and fixation, then been counted after staining with 0.1% crystal violet.

### Cell viability assay

MTT assay (Roche Diagnosis, Indianapolis, IN) was performed to determine cell viability. A549 and H322 cells were seeded (about 2×10^3^ cells per well) in 96-well plates, then transfected with siYBX1, control siRNA or PBS at the indicated doses. The cell viability was determined at 24h and 48h respectively. The effect on cell viability was assessed as the percent cell viability compared with control-PBS group, which were arbitrarily assigned 100% viability.

### Apoptosis assay

Apoptosis was measured using the Annexin V- FITC Apoptosis Detection Kit (Nanjing KeyGEN Biotech. CO, LTD). A549 and H322 cells (10^5^ cells) were respectively transfected with siYBX1, control siRNA for 48h and harvested in tubes. The experimental procedure was followed by explanatory memorandum. Briefly, different treatments of cells were collected after trypsin without ethylene diamine tetraacetic acid (EDTA) digestion and washed once with cold PBS, then co-stained with FITC-labeled annexin V and propidium iodide (PI). Stained cells were analyzed using FACS Accuri C6 (Genetimes Technology Inc.).

### Cell cycle analysis

A549 and H322 cells (10^5^ cells) were respectively transfected with siYBX1, control siRNA or PBS for 48h and harvested in tubes. Then cells were washed with PBS twice and fixed with 75% ethanol in PBS overnight. The cells were pellet by centrifugation at 1,000 rpm at 4°C for 10 minutes and incubated with RNase and PI at 37°C for 20 min. Stained DNA was sorted by FACS Accuri C6 and analyzed by using FlowJo 7.6 software.

### Wound healing assay

Cell migration was performed by scratch assay. The A549 and H322 cells were transfected with si YBX1, control si RNA or PBS when cells grown to approximated full confluence, subsequently scraped with a 100 ul pipette tip to bring the sterile wound gaps, the spacing of gap was photographed by inverted microscope at 0h and 48h.

### Xenograft mouse model and tumor processing

Twelve female nude mice (4–6 weeks old, 16-18g weigh) were managed at SPF Laboratory Animal Center at Dalian medical university in strict accordance with the guidelines by the U.S. National Institutes of Health Guide for the Care and Use of Laboratory Animals. To assess the effect of siRNA-YBX1 on lung adenocarcinoma growth in vivo, the nude mice were randomly divided into 2 groups (control siRNA group, n=6; siRNA-YBX1 group, n=6) and injected 2X10^6^ cells (A549) subcutaneously near the axillary fossa. Two weeks later when tumor volume reached about 20 mm^3^, the compound of control siRNA or siYBX1 and liposome dissolved in 5% glucose was delivery into tumor twice a week for 3 weeks. Then all mice were terminated with ether anesthesia inhalation and humanely sacrificed. The tumor volume (V = (width^2^ × length)/2) and body weights were recorded. Tumor specimens were fixed in formalin and embedded in paraffin or directly stored at −80°C. Procedure of hematoxylin-eosin staining (H&E staining) was referring to our previously published article [35]. Expression of YBX1, CDC25a, Ki67 and cleaved caspase 3 were investigated using IHC staining, the experimental procedure was performed as above. (antibody of Ki67 and cleaved caspase 3 diluted 1:200 and 1:100 in PBS containing 2% goat bovine serum respectively).

### Statistical analysis

Student's t-test or analysis of variance (ANOVA) was used to compare the values of the test and control samples in vitro and in vivo. The associations between YBX1 or CDC25a expression and categorical variables were compared by Pearson chi-squared test. Survival curves were calculated using the Kaplan Meier method. The log-rank test was used to analyze overall survival (OS) time between different clinicopathological factors in lung adenocarcinoma. Univariate and multivariate analysis was performed using the Cox regression model. Data was analyzed by the SPSS 20 software (Inc, Chicago, IL). Values of p<0.05 were considered statistically significant difference.
